# Digitalisierte Gesundheitsversorgung im Jahr 2030 – ein mögliches Szenario

**DOI:** 10.1007/s00103-021-03416-8

**Published:** 2021-09-17

**Authors:** Jan Benedikt Brönneke, Julia Hagen, Philipp Kircher, Henrik Matthies

**Affiliations:** health innovation hub (hih) des Bundesministeriums für Gesundheit, Torstr. 223, 10115 Berlin, Deutschland

**Keywords:** Digitalisierung, Digitale Gesundheitsanwendungen, Digitale Medizinprodukte, Individualisierte Gesundheit, Datennutzung, Digitalization, Digital health applications, Digital medical devices, Individualized health, Data use

## Abstract

Aufgrund der schnell voranschreitenden Digitalisierung wird sich auch die Gesundheitsversorgung in den nächsten Jahren stark verändern. In Deutschland wurden durch neue gesetzliche Rahmenbedingungen bereits die Weichen z. B. für die elektronische Patientenakte (ePA), das E‑Rezept und die Einbindung digitaler Gesundheitsanwendungen (DiGA) gestellt. Das neue Fast-Track-Verfahren des Bundesinstituts für Arzneimittel und Medizinprodukte (BfArM) zur Bewertung der Erstattungsfähigkeit von DiGA ist ein wichtiger Schritt, dem in den nächsten Jahren weitere folgen werden.

Der vorliegende Beitrag beschreibt anhand eine Zukunftsszenarios für das Jahr 2030, welche gesetzlichen, technischen und alltagspraktischen Veränderungen sich bis dahin ergeben haben könnten. Im Jahr 2030 könnte die Gesundheitsversorgung in individualisiert-integrierten Behandlungspfaden organisiert sein, die den Versicherten umfassende Begleitung bieten. Interoperable digitale Komponenten könnten strukturierte Daten z. B. für Forschungszwecke zur Verfügung stellen. Datenschutzangst könnte der Vergangenheit angehören, wenn das Datenschutzrecht reformiert und harmonisiert wird sowie neue Einwilligungsverfahren für PatientInnen entwickelt werden. Neue Berufsfelder könnten sich etablieren und der Marktzugang für innovative digitale Medizinprodukte weiter verbessert werden.

Ein weiterer wichtiger Aspekt, der dazu beitragen kann, das Potenzial der digitalen Gesundheitsversorgung auszuschöpfen, ist die Schaffung eines europäischen Datenraums auf Basis einer technischen Infrastruktur, die hohe ethische und soziale Standards wahrt. Aktive Maßnahmen seitens der Gesetzgeber können die notwendigen Voraussetzungen dafür schaffen, dass Innovationen zugunsten der PatientInnen Eingang in das System finden und das deutsche Gesundheitssystem dem fortschreitenden medizintechnischen Wandel gerecht wird.

## Abstract (en)

Due to the rapidly advancing digitalization, healthcare will also change significantly in the next few years. For example, in Germany, new legal framework conditions have already set the course for the electronic patient record (ePA), the e‑prescription, and the integration of digital health applications (DiGA). The new fast-track procedure of the Federal Institute for Drugs and Medical Devices (BfArM) for evaluating the reimbursability of DiGA is an important step that will be followed by others in the coming years.

This article uses a future scenario for the year 2030 to describe the legal, technical, and practical changes that could occur by then. In 2030, healthcare could be organized in individual and integrated treatment pathways that offer comprehensive support to the insured. Interoperable digital components could, for example, make structured data available for research purposes. Doubts concerning data protection could become a thing of the past if data protection law is reformed and harmonized and new consent procedures for patients are developed. New professional fields could become established and market access for innovative digital medical products could be further improved.

Another important aspect that can help to exploit the potential of digital healthcare is the creation of a European data space based on a technical infrastructure that upholds high ethical and social standards. Active measures on the part of legislators can create the necessary conditions for innovations to be incorporated into the system for the benefit of patients and for the German healthcare system to be able to cope with the ongoing changes in medical technology.

## Einleitung

Das Jahr 2030 ist zum gegenwärtigen Zeitpunkt knapp 9 Jahre entfernt, ein Zeitraum, der nach Maßstäben der digitalen Technologien eine Ewigkeit bedeutet [1]. Wie die Gesundheitsversorgung dann aussehen wird, lässt sich nur erahnen. Dennoch lassen sich große Trends identifizieren, die sich mit hoher Wahrscheinlichkeit bis 2030 fortsetzen werden und aus medizinischer Sicht auch wünschenswert sind. Aus Sicht der Versicherten sind hierbei 2 Trends hervorzuheben: die Fundierung von diagnostischen und therapeutischen Methoden auf Daten verschiedener Provenienz sowie die auf diesen Daten und Methoden basierende individualisierte Gesundheitsversorgung, welche (zeitliche und inhaltliche) Unabhängigkeit von punktuellen Arztbesuchen oder Krankenhausaufenthalten schafft.

Neue Gesetzesinitiativen haben in Deutschland bereits die Weichen z. B. für die elektronische Patientenakte (ePA), das E‑Rezept, die Telemedizin und die Einbindung digitaler Gesundheitsanwendungen (DiGA) gestellt. Das neue Fast-Track-Verfahren des Bundesinstituts für Arzneimittel und Medizinprodukte (BfArM) zur Bewertung der Erstattungsfähigkeit von DiGA durch die gesetzliche Krankenversicherung (GKV) war im Jahr 2020 ein wichtiger Schritt, dem in den nächsten Jahren weitere folgen werden.

Der Aufsatz wagt den Blick „zurück in die Zukunft“ auf einen Ausschnitt der Gesundheitsversorgung, geprägt durch die genannten Trends. Aus der Perspektive des fiktiven Gesundheitssystems im Jahre 2030 wird auch auf die Entwicklungen der bis dahin vergangenen Jahre zurückgeblickt und beschrieben, welche regulatorischen Änderungen zu der angenommenen Versorgungstransformation führen könnten.

Der Beitrag zeichnet zunächst das utopische Szenario, nimmt sodann an, dass individualisiert-integrierte Versorgungspfade den wesentlichen strukturellen Wandel bedeuten könnten, und beschreibt anschließend, welche zentralen regulativen Reformen in den Bereichen Datennutzung, Berufsausbildung und Medizinprodukte hierzu maßgeblich wären. Im Ausblick werden Handlungsempfehlungen aus dem Gedankenexperiment zurück in die Gegenwart übersetzt.

## Gesundheitsversorgung im Jahr 2030

Nele wird wie jeden Morgen sanft von ihrem Smart Device geweckt. Ein kurzer Blick auf das Display (Wetter: 17 °C, kein Regen; ePA: lange Tiefschlafphase, Puls o. k., leicht erhöhte Temperatur; Kalender: nächster Termin 08:30 Uhr Teambesprechung in der Praxis) lässt sie aus dem Bett federn.

Dass ihre Tiefschlafphasen sich dank der Schlaf-DiGA so schnell und nachhaltig gebessert haben, freut sie sehr. Aber erhöhte Temperatur schon am zweiten Tag in Folge? Der hauchdünne Multisensor auf ihrem Handgelenk scheint aber einwandfrei zu funktionieren. Ein Blick auf ihr Health-Dashboard in der ePA zeigt den Verlauf ihrer aktuell kritischen Gesundheitsparameter. Und hinter den heutigen Temperaturwerten ist bereits ein Action-Item vermerkt: „Abklärung Infekt“ mit einem vorgeschlagenen Termin heute Nachmittag bei ihrem Hausarzt. Den bestätigt sie per Click und er erscheint mit einem kurzen Aufblinken in ihrem Kalender. Ihr Hausarzt hat vor Kurzem auf die volldigitale Infrastruktur eingeschwenkt.

Seit einigen Monaten arbeitet Nele im medizinischen Versorgungszentrum als „Digital MFA“, also als Medizinische Fachangestellte mit Digitalisierungsspezialisierung. Auf dem Weg zur Arbeit gönnt sie sich wieder eines der köstlichen Croissants aus der 3‑D-Druck-Backautomatenbäckerei. Ihre Blutwerte hat sie seit Nutzung ihres Gesundheitsdashboards so gut im Griff, dass sie sich bei der Ernährung überhaupt nicht mehr zurückhalten muss. Nele denkt kurz zurück an ihre Abiturzeit in den 2020er-Jahren: Die ePA wurde damals gerade eingeführt, DiGA gab es vereinzelt, doch ohne automatische Anbindung an die ePA, ohne automatisierte, KI-basierte Diagnose- und Therapieunterstützungen. Das waren damals relativ einfache Anwendungen, weit entfernt von den digitalen Gesundheitsbegleitern, die heute in den Digital Health Valleys in ganz Deutschland und Europa für den Weltmarkt entwickelt werden. Zum Glück hatte damals der DiGA-Fast-Track den Weg geebnet, dass in Deutschland und Europa ein Umfeld geschaffen wurde, in dem DiGA zeigen konnten, wie effektiv und effizient sie, eingebettet in den Alltag der PatientInnen, die Gesundheitsversorgung ergänzen und verbessern können.

Zum Launch des Fast-Tracks konnten wesentliche Gesundheitsdaten noch nicht einfach aufbereitet und den PatientInnen zur Verfügung gestellt werden. Arzttermine musste man noch telefonisch vereinbaren und ihr Job als Digital MFA wurde lediglich als theoretische Option diskutiert. Überfüllte Wartezimmer, in denen eine Mehrheit der PatientInnen lediglich eine verlängerte Krankschreibung oder eine neue Verordnung benötigt, sind heute nicht mehr denkbar, genau wie das „Abarbeiten“ von PatientInnen im 6‑ bis 7‑Minuten-Takt.

Nele kann sich gar nicht mehr vorstellen, wie damals die gesamte Gesundheitsversorgung durch das Nadelöhr der hausärztlichen Sprechstunde vor Ort gefädelt werden musste. Die Akademisierung bestehender Berufe und die Schaffung neuer Rollen – z. B. die der Digital MFA und des Datentreuhänders – haben Abhilfe geschaffen: Die verteilten Rollen und die enge Kooperation erleichtern den Zugang zur Versorgung auch in der Fläche und ermöglichen es PatientInnen – digital unterstützt – schnell die richtigen Versorgungspfade zu finden. Wie absurd es heute doch anmutet, dass vielen erst durch die Coronapandemie der Mehrwert der Digitalisierung und der Verfügbarkeit guter Daten bewusst wurde.

## Individualisiert-integrierte Behandlungspfade

Heute, im Jahr 2030, richtet sich die Behandlung individuell an der Situation der Versicherten und PatientInnen aus. Auch wenn wie in den 2020er-Jahren weiterhin unterschiedliche Vergütungsregelungen in den Sektoren des Gesundheitswesens bestehen, so wurden die Sektorengrenzen doch insofern erfolgreich überwunden, als dass Anreize zur verstärkten Zusammenarbeit auf Basis von patientenindividuellen outcome-basierten Vergütungsmodellen geschaffen wurden. Diese stellen im Vergleich zu den herkömmlichen Pauschalen und individuell abrechenbaren Leistungen das Gros der Vergütung dar. Grundlage für diese umfassende Reform waren Überlegungen zu sogenannten Capitation-Modellen (pauschale Vergütung von Gesundheitsleistungen) und regionalen Gesundheitsbudgets. Diese Ansätze wurden durch die digitale Unterstützung und die bessere Verfügbarkeit von Daten, u. a. für die Bewertung von Outcomes, erfolgreich weiterentwickelt und umgesetzt [2].

Bestandteil der Bewertung der patientenindividuellen Outcomes sind auch Struktur- und Verfahrensverbesserungen, die durch digitale Gesundheitsanwendungen etabliert werden konnten [3]. So haben z. B. die gesteigerte Gesundheitskompetenz, die Adhärenz und die leitlinientreue Versorgung als Faktoren, die zum Erfolg der Behandlung beitragen, deutlich an Relevanz gewonnen. Die Unterstützung der PatientInnen ist dabei individuell zugeschnitten. Persönliche digitale Tools klären auf, informieren und leiten an die richtige Stelle in der Versorgung. Hierfür wurde auch der Zugang zu niedrigschwelliger medizinischer Versorgung verbessert. So entwickelten sich beispielsweise die Apotheken zu Telemedizin-Hubs weiter, in denen BürgerInnen mit Unterstützung der ExpertInnen vor Ort im Telemedizin-Hub telemedizinisch erstberaten werden. Daneben finden telemedizinische Beratungen auch weiterhin im häuslichen Umfeld oder gar unterwegs statt, wie es durch die Coronapandemie fest etabliert wurde. Niedrigschwellige medizinische Unterstützung funktioniert jedoch nicht nur in einer Richtung. Nimmt beispielsweise ein chronisch kranker Patient den nächsten Vorsorgetermin nicht wahr, wird ihm proaktiv ein telemedizinisches Beratungsgespräch angeboten. Das Ziel der Therapietreue wird so gemeinsam erreicht [4].

Dank digitaler Tools und angereizt durch die capitation-basierte Vergütung, haben sich in der Gesundheitsversorgung strukturierte Behandlungspfade entwickelt. Dabei sorgen die digitalen Tools dafür, dass Versicherte und PatientInnen stets über ihren eigenen Gesundheitszustand Bescheid wissen, die nächsten Schritte kennen und im Zweifel schnell und zuverlässig mit dem „richtigen“ Leistungserbringer verbunden werden, der mit wenig Aufwand über Schnittstellen alle relevanten Daten einsehen kann. Diese digitale Lotsenfunktion setzt nicht erst am Fall der akuten Erkrankung und dem damit verbundenen Arztbesuch im ambulanten und stationären Sektor an, sondern bereits zu Hause [5] vor der Erkrankung und begleitet PatientInnen durchgehend zwischen Arztbesuchen, in der Nachsorge und chronisch Kranke sogar lebenslang. Die Erstellung persönlicher, multidimensionaler, longitudinaler Gesundheitsprofile einer jeden PatientIn ermöglicht zudem eine kontinuierliche Triage, die die Ressourcen der Leistungserbringer optimal allokiert und zu jedem Zeitpunkt die individuell benötigten Leistungen ermöglicht.

## Nutzung von Daten

Aus der Perspektive im Jahr 2030 ist es kaum noch nachzuvollziehen, wie – im wahrsten Sinne des Wortes – „unstrukturiert“ es um die 2020er-Jahre noch zuging. Selbstverständlich ist es mittlerweile Standard, dass Daten in interoperablen Formaten verarbeitet werden und medizinische Informationen entsprechend als strukturierte Daten vorliegen. Je nach Kontext können sie also zu übersichtlichen Ansichten (ähnlich einem Dokument) zusammengeführt, automatisiert interpretiert und aufbereitet dargestellt oder in andere Sprachen übersetzt werden. All das geht ohne die Abhängigkeit vom jeweiligen Anbieter (sog. Lock-in-Effekte), weil unterschiedliche Hard- und Software die Daten bedienen und mit fremden Systemen austauschen können, sodass Nutzer nicht mehr dauerhaft an ein proprietäres System gebunden sind.

Was in der Medizin als Notwendigkeit der Behandlungssicherheit begann, diffundierte alsbald auch in den Consumer-Bereich, wo etwa die Macht der großen Plattformen (z. B. soziale Medien und Messengerdienste) gebrochen werden konnte [6]. Plattformökonomische Ansätze gab und gibt es natürlich noch immer. Aber die sozialversicherungsrechtlichen Vorgaben zur Verwendung von internationalen Standards und offenen Schnittstellen haben eine Monopol- und Kartellbildung verhindert. So können Versicherte über die Software ihrer Wahl nach ihrem Wunsch Daten mit unterschiedlichen Leistungserbringern im In- und Ausland austauschen, unabhängig davon, welche technische Infrastruktur von diesen genutzt wird. Das hat eine Modularisierung [7] von Patientenverwaltungssystemen erlaubt, die den Wettbewerb auf dem Markt der komplexen Krankenhaus- und Praxisinformationssysteme in besonderer Weise verändert hat. Statt sich auf die Entwicklung von All-in-one-Lösungen zu versteifen, konnten Softwareentwickler spezialisierte Module entwickeln, die je nach den Bedürfnissen eines Leistungserbringers angeboten werden können. Interoperabilität war die technische Basis. Ein echter Durchbruch aber folgte erst etwas später durch die Vereinheitlichung von Datenschutzanforderungen.

Die *Deutsche Datenschutzangst* der ersten beiden Jahrzehnte des 21. Jahrhunderts war in der internationalen Gesundheitsszene ein geflügeltes Wort geworden. Die Diskussion war zu dem Zeitpunkt einseitig geprägt von möglichen Datenmissbrauchsszenarien. Nur ein „nichtexistentes Datum“ war ein „gutes Datum“. Es brauchte mehrere Anläufe und die Coronapandemie als Katalysator, um das Datenschutzrecht ernsthaft zu reformieren und international zu harmonisieren – ein Ziel, das die Datenschutz-Grundverordnung (DSGVO) ursprünglich verfolgt, aber leider nie erreicht hatte, weder im Bereich der Forschung [8] noch im Bereich der Krankenhausbehandlung [7]. Erst durch die Coronapandemie wurde vielen klar, dass Daten auch Leben retten, die Gesundheitsversorgung verbessern und PatientInnen in die Lage versetzen können, sich selbstständiger aktiv um ihre Gesundheit zu kümmern [9].

Damals wurden die ersten Ansätze zu einer breiten Einwilligung (Broad Consent) entwickelt [10]. Diese ermöglichten erstmalig die Festlegung des grundsätzlichen Umgangs mit Gesundheitsdaten über konkret benannte Einzelzwecke hinweg. Der Ansatz ließ zu, dass BürgerInnen frühzeitig über die Nutzung ihrer Daten entscheiden, lange bevor bei ihnen Diagnosen, Therapien oder gar schwerwiegende Eingriffe anstehen, so wie dies bei Patientenverfügungen oder der Organspende schon lange akzeptiert war. Das Zusammenspiel aus Broad-Consent-Ansätzen, intelligenten Einwilligungstools, dem zunehmend ausgebauten Forschungsdatenzentrum und der Dateninfrastruktur von Gaia‑X [11] trug dazu bei, dass Europa zu einem führenden Ökosystem für Gesundheitsdatennutzung und -forschung werden konnte, ohne die Selbstbestimmung der Individuen einzuschränken. Dieser neue europäische Standard fand weltweit Nachahmer und trug dazu bei, Europas Rolle als florierendes Ökosystem für digitale Medizin und Forschung weiter auszubauen.

Die Coronapandemie führte immer wieder den Bedarf an schnell verfügbaren, verlässlichen Daten vor Augen. Das führte zu einer Revision des Datenschutzrechts und zu der Einsicht, dass es weder erforderlich geschweige denn sinnvoll gewesen war, in jeder Regelungsmaterie landesspezifisch gesonderte datenschutzrechtliche Zulässigkeitsbeschränkungen und divergierende Anforderungen an technische und organisatorische Maßnahmen zu formulieren. Es wurde klar, dass Rechtskonformität nur dann erreicht wird, wenn einheitliche und verständliche Anforderungen für alle Beteiligten gelten. Dabei siegte die Erkenntnis, dass Datenschutz- und Datensicherheit faktisch signifikant verbessert werden können, wenn praktikable Standards eingesetzt werden, anstatt abstrakte Anforderungen zu formulieren, die erhebliche Auslegungsspielräume und damit einerseits Umgehungmöglichkeiten und andererseits Rechtsunsicherheiten beinhalten [12].

Der 1. wesentliche Schritt war dabei die weitgehende Aufhebung der vielen Möglichkeiten der EU-Mitgliedsstaaten, durch nationales Recht von der DSGVO abzuweichen. Nur im Fall von explizit zu begründenden Besonderheiten der Verfassung des jeweiligen Mitgliedsstaats durfte von diesen sog. Öffnungsklauseln noch Gebrauch gemacht werden. Gleiches gilt seither für das Datenschutzrecht der Religionsgemeinschaften. In einem 2. Schritt erfolgte die Schaffung einer Gesetzgebungskompetenz des Bundes für den Datenschutz. Das große föderale Durcheinander war damit bereits aufgeräumt. Der 3. wesentliche Teil der Reform war – nach anfänglichem Widerstand [13, 14] – die Änderung der Datenschutzaufsichtsstrukturen innerhalb Deutschlands. So wurde die Datenschutzkonferenz unter Leitung des Bundesbeauftragten für den Datenschutz und die Informationsfreiheit (BfDI) als zentrale Behörde weiterentwickelt. Den Behörden in den verschiedenen Ländern wurden bestimmte Zuständigkeiten nach Themengebieten zugeteilt – statt in jedem Land redundante, aber jeweils nur dünn besetzte Strukturen vorzuhalten, gibt es seither 16 Datenschutzfachbehörden.

Es dauerte aber bis nach dem „Schrems-IV-Urteil“ 2025, in dem der Europäische Gerichtshof das „Super-Safe-Privacy-Harbor-Shield-System“ gekippt hatte, dass auch die USA sich einer gemeinsamen Datenschutzgesetzgebung unterwarfen. Ein neuer „Personal-Information-Processing-Act“ (PIPA) hat die DSGVO abgelöst und findet seither schon in über 120 Ländern Anwendung. Wesentliche Änderung: Statt davon auszugehen, dass die Verarbeitung eines Datums im Grundsatz unzulässig ist, hat man den Fokus auf die Absicherung der Verarbeitung von personenbezogenen Informationen gelegt. Wie sensibel eine Information ist, wird kontextabhängig bestimmt und nicht etwa aufgrund der Tatsache, dass ein Gesundheitsdatum vorliegt. Dabei wurde das Schutzziel der Verfügbarkeit von Informationen im Interesse der betroffenen Person als vorrangig im Verhältnis zur Vertraulichkeit gewichtet [9].

Interoperable Daten aus DiGA, ePA, E‑Rezepten und Wearables werden seither entweder von einem Datentreuhänder nach dem Data-Governance-Gesetz [15] oder im einheitlichen Datendashboard der ePA verwaltet. Hier werden Consent-Management, Datenschutzerklärungen und die Wahrnehmung von Betroffenenrechten u. v. m. an einem Ort zusammengeführt. Die Herausgabe von Realdaten ist für die Forschung allerdings nur noch in wenigen Fällen erforderlich. Techniken zur Synthetisierung von Datensätzen werden auf Basis von Echtdaten vom Forschungsdatenzentrum erstellt und ForscherInnen zur Verfügung gestellt. Ein nicht unerheblicher Teil erster Validierungsphasen von Forschungsvorhaben kann daher innerhalb weniger Minuten durchgeführt werden, statt – wie damals – nach mehrjährigen Anträgen auf Datenzugangsgewährung. Ein willkommener Nebeneffekt: Die Standardisierung von Daten macht es möglich, Daten dezentral zu halten und im Bedarfsfall dennoch ad hoc zusammenzuführen.

## Neue Berufsfelder und -ausbildungen

Komplementär zur digital unterstützen, integrierten Versorgung entwickelten sich Berufsbilder und Rollen im Gesundheitssystem weiter. Dies resultierte nicht nur aus den veränderten Versorgungstrukturen, sondern auch aus einem Fachkräftemangel. Als Ergebnis wurde die Rolle der *Digital MFA*, als Weiterentwicklung der Community Nurse [16], verstärkt und ihr Aufgabenspektrum ausgeweitet (vgl. u. a. VERAH [17]).

Die Digital MFA ist die erste Anlaufstelle vor Ort. Bei der Ersteinschätzung von Symptomen wird sie von digitalen Expertensystemen unterstützt. Die Praxisräume der Digital MFA dienen zudem als Telemedizin-Hub, über den ärztliche Expertise hinzugezogen werden kann. Auch spezialisierte fachärztliche Expertise kann dadurch ortsunabhängig eingeholt werden. Vor Ort unterstützt die Digital MFA bei körperlichen Untersuchungen und der Erhebung von Vitalparametern. Die Ergebnisse werden über die ePA verzögerungsfrei den sonstigen an der Versorgung beteiligten Leistungserbringern zur Verfügung gestellt.

Aufgrund der veränderten Rollen im Gesundheitswesen und der stärkeren Zusammenarbeit zwischen unterschiedlichen Gesundheitsberufen wurde die Anpassung des Berufsrechts notwendig. Dabei wurde insbesondere eine Modularisierung der Aus- und Weiterbildung vorgenommen, sodass Digital MFA auch Zusatzqualifikationen erlangen können, um gewisse ärztliche Leistungen selbst zu erbringen.

Obwohl in den 2020er-Jahren bereits allen klar war, dass BürgerInnen selbst die Cookie-Einwilligungen auf Webseiten kaum noch inhaltlich durchdringen können, dauert es noch einige Zeit, bis sich der Beruf des *Datentreuhänders* entwickelte. In einer datenarmen Medizin der Vergangenheit hatte es genügt, wenn ÄrztInnen über die Erkrankungen ihrer PatientInnen einfach nicht sprachen.^1^ Der Fokus lag daher lange auf der Datenminimierung und Vertraulichkeit von Informationen. Neben einer ärztlichen Schweigepflicht wurde folglich etwa das Sozialgeheimnis für die Leistungsträger des Sozialversicherungssystems geschaffen [19]. Doch je mehr der Wert guter Daten für eine bessere Forschung und Versorgung entdeckt wurde, desto mehr verlagerte sich der Fokus von Verschwiegenheitsverpflichtungen hin zum Datenschutz. Zunächst erfolgte die Umsetzung des Datenschutzes weitgehend unstrukturiert in unüberblickbaren Verästelungen. Die Menge der verarbeitenden Stellen, die unterschiedlichen rechtlichen Regelungen und die schiere Menge an Daten sowie die Implikationen ihrer Verwendung wurden ohne spezielle Fachkunde bald schon nicht mehr handhabbar. Mit dem Patientendaten-Schutz-Gesetz (PDSG) hatte der Gesetzgeber für die Verwendung der ePA bereits vorgesehen, dass Personen, die diese nicht nutzen konnten oder wollten, einen Vertreter bestimmen könnten.

Auf anfängliche Kritik an der Rechtskonformität einer solchen Lösung [20] entwickelten sich schnell Ideen zur Schaffung eines neuen Vertrauensberufs, dem des Datentreuhänders [15]. Speziell geschulte, akkreditierte und zur Vertraulichkeit verpflichtete Personen konnten sich die Aufgabe der Verwaltung persönlicher Daten von einer natürlichen Person übertragen lassen. Ob die sicher verschlüsselte Hinterlegung wichtiger Dokumente oder die Verwaltung von Zugriffsrechten auf Daten der ePA – der Datentreuhänder wurde schnell eine der wesentlichen Vertrauenspersonen von BürgerInnen. Auch wenn auf dem Land häufig keine HausärztInnen mehr vor Ort waren und durch anonyme Telemedizinanbieter ersetzt wurden, waren Daten nicht mehr verloren oder nur mit besonderen technischen Fähigkeiten zugänglich. Neben SteuerberaterInnen, RechtsanwältInnen und NotarInnen entwickelte sich der verkammerte Beruf des Datentreuhänders schnell zu einem der elementaren Vertrauenspfeiler der Gesellschaft. Nicht nur half er die digitale Kluft (Digital Divide) zu überbrücken, er sorgt nun für eine umfassende Entlastung der BürgerInnen in allen Datenangelegenheiten – auch über den Gesundheitsbereich hinaus.

## Marktanforderungen und -bedingungen für innovative Softwareprodukte

Die Erkenntnis, dass personalisierte Medizin auf strukturierte, interoperable Daten angewiesen ist, zeitigte nicht nur eine neue Regulierung der vorhandenen Berufsbilder, der Datenerhebung und des Datengebrauchs, sondern führte auch zu weitreichenden Novellierungen der Medizinprodukteregulierung sowie zur Weiterentwicklung des GKV-Zugangs für innovative digitale Produkte.

Bereits kurz nach Geltungsbeginn der Medical Device Regulation (MDR) wurde offenbar, dass das mit der MDR weiterentwickelte System der Konformitätsbewertung für die Bewertung von Stand-alone-Software als Medizinprodukt (Software as a Medical Device, SaMD) weitgehend ungeeignet war, insbesondere in Hinblick auf digitale Lösungen mit besonders niedrigem oder besonders hohem Risiko: Zum einen gelang es nicht, niedrigschwellige und risikoarme digitale Angebote zur Sammlung, Strukturierung und Auswertung von patienteneigenen Daten, wie etwa digitale Tagebücher mit Erinnerungsfunktion und basaler Datenauswertung zur grafischen Datendarstellung, den Marktzugang auf Basis adäquater Anforderungen zu ermöglichen.^2^ Digitale Lösungen, die in allen Gesundheitssystemen Europas als eine Säule der patientengesteuerten Datengenerierung und Auswertung für die jeweiligen elektronischen Patientenakten^3^ angesehen werden, wurden vielfach durch den Umstand hoher finanzieller Aufwände der Konformitätsbewertung und zugleich relativ geringer Vergütung gehemmt.

Zum anderen konnte auch kein transparenter und sicherer Marktzugang für komplexe und mit hohem Risiko einhergehende Technologien auf Basis von selbstlernenden KI-Algorithmen geschaffen werden.^4^ Die mangelnde Flexibilität der MDR^5^ führte dazu, dass Hersteller von SaMD weniger performante Produkte auf den europäischen Markt brachten und insbesondere neue Funktionen dieser SaMD mit erheblicher Verzögerung bereitgestellt wurden^6^, obwohl die Ausgangssituation im Jahr 2021 zunächst durchaus anderes vermuten ließ.^7^ Hiervon waren auch die Hersteller klassischer, nicht digitaler Medizinprodukte betroffen, deren Produkte häufig in nur eingeschränkten Versionen auf den europäischen Markt gebracht werden konnten. Zugleich wurde offenbar, dass die heterogenen Regelungen für die Vergütung von digitalen Produkten in den mitgliedsstaatlichen Gesundheitssystemen sowie die starren Strukturen der Nutzenanalyse den Marktzugang für viele Produkte unattraktiv gestalteten.

Auf europäischer Ebene wurde als Konsequenz dieser Umstände in der European Medicines Agency (EMA) eine neue Abteilung für digitale Medizinprodukte eingesetzt, die in Zusammenarbeit mit der Medical Device Coordination Group (MDCG), den Interessensgemeinschaften der Benannten Stellen und den Normierungsinstituten die gemeinsamen Spezifikationen für digitale Medizinprodukte festlegte und nun beständig weiterentwickelt.

Zugleich begann das European Network for Health Technology Assessment (EUnetHTA) mit der Entwicklung eines einheitlichen europäischen Rahmens für die Nutzenbewertung von digitalen Medizinprodukten. Durch die Einbindung der EMA in diesen Prozess wurde dabei besonderes Augenmerk auf die Vermeidung von Friktionen und Inkonsistenzen zwischen der medizinprodukterechtlichen Konformitätsbewertung und den national geprägten Systemen zur Bewertung von Gesundheitstechnologien (HTA) gelegt, sodass schließlich ein relativ harmonisierter Weg für digitale Medizinprodukte von der Markteinführung bis hin zur Anerkennung der Produkte in den nationalen Gesundheitssystemen entstand. Herausragendes Merkmal dieses sogenannten Dynamic Digital Pathway ist die Abkehr vom statischen Modell der punktuellen Überprüfung der Sicherheit, Leistungsfähigkeit und des Nutzens auf Basis von Studien, hin zu einer – wiederum digital gesteuerten – dynamischen und kontinuierlichen Prüfung und Überwachung digitaler Gesundheitstechnologien.

Deutschland setzte als erstes europäisches Land den Vorschlag des Dynamic Digital Pathway um. In diesem Rahmen wurde der Begriff der DiGA um Medizinprodukte der medizinprodukterechtlich höheren Risikoklassen IIb und III erweitert und für diese prozessual angepasst: Innerhalb von 6 Monaten entscheidet das BfArM nun über deren Erstattungsfähigkeit. Das umfasst auch eine Prüfung der im Vergleich zu den DiGA niedriger Risikoklassen höheren Evidenzanforderungen durch das Institut für Qualität und Wirtschaftlichkeit im Gesundheitswesen (IQWiG). Fester Bestandteil der Aufnahme in das DiGA-Verzeichnis wurde die kontinuierliche Überprüfung der Sicherheit und des positiven Versorgungseffekts auf Basis der in den DiGA erzeugten und in Registern des BfArM aggregierten Daten.^8^ Diese Daten wurden aber nicht nur für die Bewertung der jeweiligen DiGA eingesetzt, sondern – PIPA-konform – über interoperable Schnittstellen auch der Forschung zugänglich gemacht, der damit erstmals in Deutschland eine datenschutzkonforme und umfassende Datenbasis für Echtweltstudien zur Verfügung steht.

Die DiGA nehmen damit und auf Basis der grundsätzlich interoperabel vorliegenden Daten im Jahr 2030 eine maßgebliche Schnittstellenfunktion zwischen verschiedenen Datenquellen ein. Sie verbinden zum Beispiel die von ÄrztInnen genutzten Medizingeräte, softwaregestützte Hilfsmittel und Implantate sowie Consumer Technology. Damit generieren sie – sowohl unmittelbar als auch mittelbar über die ePA – Erkenntnisse für die PatientInnen und ihre behandelnden ÄrztInnen im jeweiligen Behandlungsfall und können darüber hinaus Daten für die universitäre und industrielle Forschung bereitstellen.

## Zurück im Jahr 2021 – Ein Ausblick

Digitale Gesundheitstools können im Jahr 2030 für Versicherte und PatientInnen in Deutschland einen heute kaum zu erfassenden Nutzen stiften. Ob dies gelingt, hängt maßgeblich von rechtlichen Weichenstellungen ab, die die notwendigen Schritte zu einer personalisierten – und damit besseren – Medizin erlauben: Ausgehend vom Nutzen für die Versicherten und PatientInnen ist der personalisierten Gesundheitsversorgung auf Basis von verfügbaren und sinnvoll nutzbaren Daten und unter Zuhilfenahme hierfür ausgebildeter Leistungserbringer der Weg zu ebnen. Dies bedarf Anpassungen der Berufsbilder, des Datenschutzrechts und letztlich auch der Regulierung des Markzugangs digitaler (Medizin‑)Produkte. Die aufgezeigten Aspekte entsprechender Weichenstellungen sind nur wenige neben vielen weiteren, zuvorderst etwa der Schaffung eines europäischen Datenraums auf Basis einer technischen Infrastruktur, die die hohen ethischen und sozialen Standards in Europa einhält.

Der DiGA-Fast-Track hat gezeigt, dass in dem hoch regulierten Bereich der Gesundheitsversorgung, insbesondere dem GKV-Recht, nur aktive Maßnahmen seitens der Gesetzgeber die notwendigen Voraussetzungen dafür schaffen können, dass Innovationen zugunsten der PatientInnen Eingang in das System finden: Angesichts der vorhandenen Widerstandskraft (Resilienz) des deutschen Gesundheitssystems kann ein „beobachtendes Warten“ dem fortschreitenden medizintechnischen Wandel nicht gerecht werden. Ob die hier skizzierten Wege die richtigen sind, ist diskutabel. In den futurologisch aufgezeigten Aspekten besteht aber ohne Zweifel dringender Handlungsbedarf, wenn die Gesundheitsversorgung nicht von vitalen technischen Mitteln der zukünftigen Medizin abgeschnitten sein soll.

## Footnotes

1 Als „ars muta“ wurde die verschwiegene Kunst der Medizin im antiken Rom bezeichnet [18].

2 Diese risikoarmen digitalen Produkte waren aufgrund der restriktiven Auslegung der Regel 11 im Sinne der Medical Device Coordination Group (MDCG) Guidance stets als mindestens Klasse IIa-Produkt eingestuft worden ([3, 21]).

3 Technische und praktische Möglichkeiten hierfür bestanden bereits im Jahr 2019 [22], sofern konsequent interoperable Standards genutzt wurden [23].

4 Dies resultierte aus dem Umstand, dass nach MDR die Validierung und Verifizierung des zugrunde liegenden Algorithmus und dessen Prüfung durch Benannte Stellen für die Konformität entscheidend waren. Der Algorithmus durfte sich nach der Konformitätsbewertung damit kaum mehr verändern [24].

5 Trotz seiner Unzulänglichkeiten wurde im Jahr 2020 der risikobasierte Ansatz der MDR als „Blaupause“ für die grundsätzliche Regulierung von KI-Systemen herangezogen [25].

6 Diese waren beispielsweise in den USA stets früher verfügbar, da die U.S. Food and Drug Administration (FDA) bereits im Jahr 2022 mit einem entsprechenden regulatorischen Rahmen einen transparenten und sicheren Zugang geschaffen hatte [26].

7 Im Jahr 2021 konnte in Europa eine größere Anzahl „zugelassener“ Systeme als in den USA ausgemacht werden [27]. Diesen Vorsprung bei statischen Algorithmen konnte die EU jedoch mit dem vermehrten Aufkommen von selbstlernenden Systemen nicht aufrechterhalten.

8 Ähnlich der anwendungsbegleitenden Datenerhebung nach § 35a Abs. 3b SGB V und orientierend an den Vorschlägen des IQWiG zur anwendungsbegleitenden Datenerhebung [28].
